# Case Report: Dengue and COVID-19 Coinfection in Thailand

**DOI:** 10.4269/ajtmh.20-1340

**Published:** 2020-12-15

**Authors:** Worapong Nasomsong, Viravarn Luvira, Danabhand Phiboonbanakit

**Affiliations:** 1 Division of Infectious Disease, Department of Internal Medicine, Phramongkutklao Hospital and College of Medicine, Bangkok, Thailand;; 2 Department of Clinical Tropical Medicine, Faculty of Tropical Medicine, Mahidol University, Bangkok, Thailand;; 3 Vibhavadi Hospital, Bangkok, Thailand

## Abstract

We report a 50-year-old Thai woman with recent travel to Denmark who presented with acute high-grade fever, vomiting, and myalgia for 1 day. Initial laboratory results revealed leukopenia, elevated aspartate transaminase, and elevated alanine transaminase. Chest radiograph showed no pulmonary infiltration. Reverse transcriptase–PCR (RT-PCR) of the nasopharyngeal swab detected SARS-CoV-2, and RT-PCR of the blood detected dengue virus serotype 2. COVID-19 with dengue fever co-infection was diagnosed. Her symptoms were improved with supportive treatment. Integration of clinical manifestations, history of exposure, laboratory profiles, and dynamic of disease progression assisted the physicians in precise diagnosis. Co-circulating and nonspecific presentations of dengue infection and COVID-19 challenge the healthcare system in tropical countries. To solve this threat, multi-sector strategies are required, including public health policy, development of accurate point-of-care testing, and proper prevention for both diseases.

## INTRODUCTION

COVID-19 exhibits various clinical manifestations, including acute undifferentiated febrile illness (AUFI).^1^ In Thailand, infection with dengue virus (DENV) is the most common cause of AUFI with epidemic potential throughout the year.^2^ Dengue and COVID-19 share clinical and laboratory features that are sometimes difficult to distinguish.^3^ Some reports have described COVID-19 cases that were initially misdiagnosed as dengue infection.^4–6^ Thus, dengue and COVID-19 might be a hazardous combination, and physicians should have raised awareness of this, especially in tropical areas. We report a 50-year-old woman diagnosed with laboratory-confirmed dengue fever and COVID-19 during the COVID-19 outbreak in Bangkok, Thailand.

## CASE REPORT

A 50-year-old Thai female flight attendant living in central Bangkok, Thailand, with no known medical illness presented with acute fever, myalgia, nausea, and vomiting for 1 day prior admission. Seven days before presentation, she had traveled to Denmark from March 20 to 23, 2020. Her vital signs revealed a body temperature of 39°C, respiratory rate of 16 breaths/minute, pulse rate of 82/minute, and blood pressure of 120/80 mmHg. She denied upper and lower respiratory tract symptoms. Her oxygen saturation at presentation was 98% on room air. Other physical examinations were unremarkable. Initial laboratory examination showed leukopenia with lymphopenia (white blood cells count, 3,700/µL and total lymphocyte count, 222/µL), normal platelet count, elevated aspartate transaminase (AST), elevated alanine transaminase (ALT), mild hyponatremia, and hypokalemia (Table 1). Chest radiography (CXR) showed no pulmonary infiltration. She was promptly isolated in the acute respiratory infection (ARI) clinic at the hospital, which isolated patients with fever or respiratory symptoms as well as encouraged appropriate personal protective equipment use among healthcare workers. She was subsequently admitted to a single-room cohort ward for COVID-19 while awaiting for laboratory result. Dengue point-of-care test (Bio Tracer Dengue Combo Rapid Card™, Bio Focus Co., Ltd, Gyeonggi-do, South Korea) showed a faint positive band for NS1-Ag and IgG. Thus, dengue real-time reverse transcriptase–PCR (RT-PCR) (VIASURE, CerTest BIOTEC, San Mateo de Gállego Zaragoza, Spain) was requested because of doubtful diagnosis, and DENV serotype 2 (DENV2) was detected. Reverse transcriptase–PCR for SARS-CoV-2 of pooled specimens from nasopharyngeal and throat swabs was positive. She was hospitalized with supportive treatment, close monitoring, and isolation. Fever resolved after hospitalization for 24 hours. In the first week of admission, the laboratory revealed progressive leukopenia with an increased lymphocyte ratio and slightly reduced platelet count. These spontaneously resolved in the second week of admission (Table 1). Her clinical symptoms improved, and she did not complain any respiratory symptoms throughout the admission for isolation. Chest radiography performed on second, third, and 10th days of admission showed no pulmonary infiltration. The patient was discharged on the 12th day after onset of illness. At telephone visits at 7 and 14 days after discharge, she had complete resolution of symptoms.

**Table 1:** Dynamic of laboratory profile in this patient

Laboratory	First day of admission	Second day of admission	Third day of admission	Fourth day of admission	Fifth day of admission	Seventh day of admission	10th day of admission
Hematocrit (%)	34	34	33	33	39	34	37
Hemoglobin (g/dL)	11.2	11.3	10.8	11	12.5	11.2	12.1
White blood cells (cell/µL)	3,700	1,500	3,200	3,100	3,200	4,400	4,400
Neutrophils (%)	89	48	67	66	40	34	42
Lymphocytes/atypical lymphocytes (%)	6	40	26	29	43	55/7	54
Total lymphocyte count (cell/µL)	259	600	832	899	1,376	2,420	2,376
Monocyte (%)	5	10	7	5	7	4	3
Eosinophils (%)	0	1	0	0	1	0	1
Basophils (%)	0	1	0	0	1	0	0
Platelets (cell/µL)	196,000	170,000	158,000	162,000	194,000	227,000	347,000
Aspartate transaminase (U/L)	107	75	49	–	71	45	45
Alanine transaminase (U/L)	120	104	81	–	136	101	100
Sodium (mEq/L)	132	–	135	134	136	–	–
Potassium (mEq/L)	3.2	–	3	4.1	4.2	–	–
Chloride (mEq/L)	96	–	101	100	99	–	–
Bicarbonate (mEq/L)	29	–	29	29	29	–	–
Lactate dehydrogenase (U/L)	295	–	286	285	230	219	–

## DISCUSSION

COVID-19 and dengue have spectrums of disease with overlap in clinical manifestations, including AUFI.^7,8^ Dengue is one of the most common tropical infectious diseases in Thailand.^2^ After the first confirmed COVID-19 case outside China was reported in Thailand in early January 2020,^9^ an outbreak occurred from March to May. Herein, we reported a confirmed case of COVID-19 accompanied by a dengue infection (without warning signs) during the early phase of the outbreak of COVID-19 in March 2020. The patient presented with only acute fever with nonspecific systemic symptoms, which made discriminating between COVID-19 and other tropical infections extremely difficult. An early COVID-19 study reported that it could initially present as AUFI, but later CXRs usually revealed abnormalities compatible with pneumonia.^1^ In the present case, no abnormality was detected in follow-up CXRs. This might be explained by either asymptomatic or mild COVID-19.^10^ However, we did not perform more sensitive computed tomography of the chest because of the absence of respiratory symptoms and as an infection control measure.^11^ Leukopenia with lymphopenia at presentation is found in either early dengue or COVID-19.^3^ In the present case, progressive leukopenia with relative lymphocytosis and presentation of atypical lymphocyte, decreased platelet count, and increased hematocrit occurred in the later days of illness. These findings in the later days of illness are compatible with the usual clinical course of dengue infection.^8,12^ In the present case, history of international travel and working in a crowded space were important clues to test for COVID-19. The higher ALT than AST in the present case, which was not the classic laboratory finding of dengue, also raised suspicion of another condition.^2^ The present case demonstrates the difficulty in distinguishing COVID-19 from dengue due to the variety and nonspecific nature of clinical manifestations despite confirmation with gold standard diagnostic tests. Notably, severe manifestations of these diseases are also difficult to discriminate. For example, pulmonary edema in severe dengue and severe pneumonia in COVID-19 shared clinical and radiological features.^13^ Nevertheless, hemoconcentration and plasma leakage syndrome are the unique manifestations of severe dengue, which can help discriminate dengue from COVID-19 in this setting.^8,12^ Some reports have raised concerns about more severe or fatal outcomes in these combinations.^14,15^ However, none of the patients classified as severe disease or reported fatal outcomes from the few previous reports, including the present case.^16,17^

Laboratory diagnosis of dengue in the setting of the COVID-19 pandemic is a greater challenge. Some reports have revealed false-positive dengue antibody in COVID-19 patients who were misdiagnosed as dengue.^4–6^ Serological cross-reactivity between dengue and COVID-19 has been reported.^18^ Thus, either dengue NS1Ag or dengue RT-PCR should be performed to confirm the diagnosis if suspected. Testing for both dengue and COVID-19 with accurate methods together with isolation of suspected cases while awaiting the results is ideal and a well-established practice in some institutes.^6^ However, this approach might overwhelm a public health system in a resource-limited tropical setting during an outbreak. During the COVID-19 outbreak in Thailand, the national policy encouraged all hospitals to establish ARI clinic to isolate and facilitate patients presenting with fever or respiratory symptoms. This clinic will provide a rapid examination of suspected COVID-19 cases separated from other patients.^19^ Free SARS-CoV-2 testing is provided for suspected COVID-19 cases meeting the Ministry of Public Health’s criteria which are updated concerning the situation.^20^ By contrast, laboratory investigations for other causes of fever in an individual patient are selected based on clinical suspicion of the physicians in charge.

In summary, COVID-19 should be included in the differential diagnosis of AUFI even if another infection has also been found. Considering clinical manifestations, history of exposure, laboratory profiles, and dynamic of disease progression together is the most important strategy for precise diagnosis. During the COVID-19 pandemic, precautionary isolation of AUFI patients should be considered to prevent the spread of infection. Nevertheless, isolation may lead to unrecognized and delayed management of dengue vascular leakage syndrome. Multi-sector strategies including public health policy, development of accurate point-of-care testing, and proper prevention for both diseases should be integrated to solve this problem.
